# Molecular mechanisms controlling asymmetric and symmetric self-renewal of cancer stem cells

**DOI:** 10.1186/s40543-015-0071-4

**Published:** 2015-10-15

**Authors:** Young Dong Yoo, Yong Tae Kwon

**Affiliations:** Protein Metabolism Medical Research Center and Department of Biomedical Sciences, College of Medicine, Seoul National University, Seoul, 110-799 Korea; Neuroscience Research Institute, Seoul National University College of Medicine, Seoul, Korea; Ischemic/Hypoxic Disease Institute, College of Medicine, Seoul National University, Seoul, 110-799 Korea

**Keywords:** Cancer stem cell, Normal tissue stem cells, Asymmetric and symmetric cell division, Fate determinants, Self-renewal, Cancer therapy

## Abstract

Cancer stem cells (CSCs), or alternatively called tumor initiating cells (TICs), are a subpopulation of tumor cells, which possesses the ability to self-renew and differentiate into bulk tumor mass. An accumulating body of evidence suggests that CSCs contribute to the growth and recurrence of tumors and the resistance to chemo- and radiotherapy. CSCs achieve self-renewal through asymmetric division, in which one daughter cell retains the self-renewal ability, and the other is destined to differentiation. Recent studies revealed the mechanisms of asymmetric division in normal stem cells (NSCs) and, to a limited degree, CSCs as well. Asymmetric division initiates when a set of polarity-determining proteins mark the apical side of mother stem cells, which arranges the unequal alignment of mitotic spindle and centrosomes along the apical-basal polarity axis. This subsequently guides the recruitment of fate-determining proteins to the basal side of mother cells. Following cytokinesis, two daughter cells unequally inherit centrosomes, differentiation-promoting fate determinants, and other proteins involved in the maintenance of stemness. Modulation of asymmetric and symmetric division of CSCs may provide new strategies for dual targeting of CSCs and the bulk tumor mass. In this review, we discuss the current understanding of the mechanisms by which NSCs and CSCs achieve asymmetric division, including the functions of polarity- and fate-determining factors.

## Review

### Introduction

Cancers are composed of a heterogeneous population of hierarchically organized and functionally diverse cells, in which cancer stem cells (CSCs) are placed at the top. CSCs are characterized by self-renewal ability to maintain their proportion in tumors and the potential to differentiate into non-tumorigenic bulk tumor cells (Schatton et al. 2008). Abundant evidence suggests that CSCs are responsible for the growth and recurrence of tumors and their resistance to chemo- and radiotherapy (Alison et al. 2011; Clevers 2011). The ratio of self-renewing CSCs and the activity of non-CSCs to de-differentiate back to CSCs have been correlated with poor prognosis and clinical outcomes of cancers (Frank et al. 2010; Pece et al. 2010). Increasing attention is drawn to the development of therapeutic strategies targeting CSCs, singly or in combination of traditional treatments targeting bulk tumor masses.

**Fig. 1:**
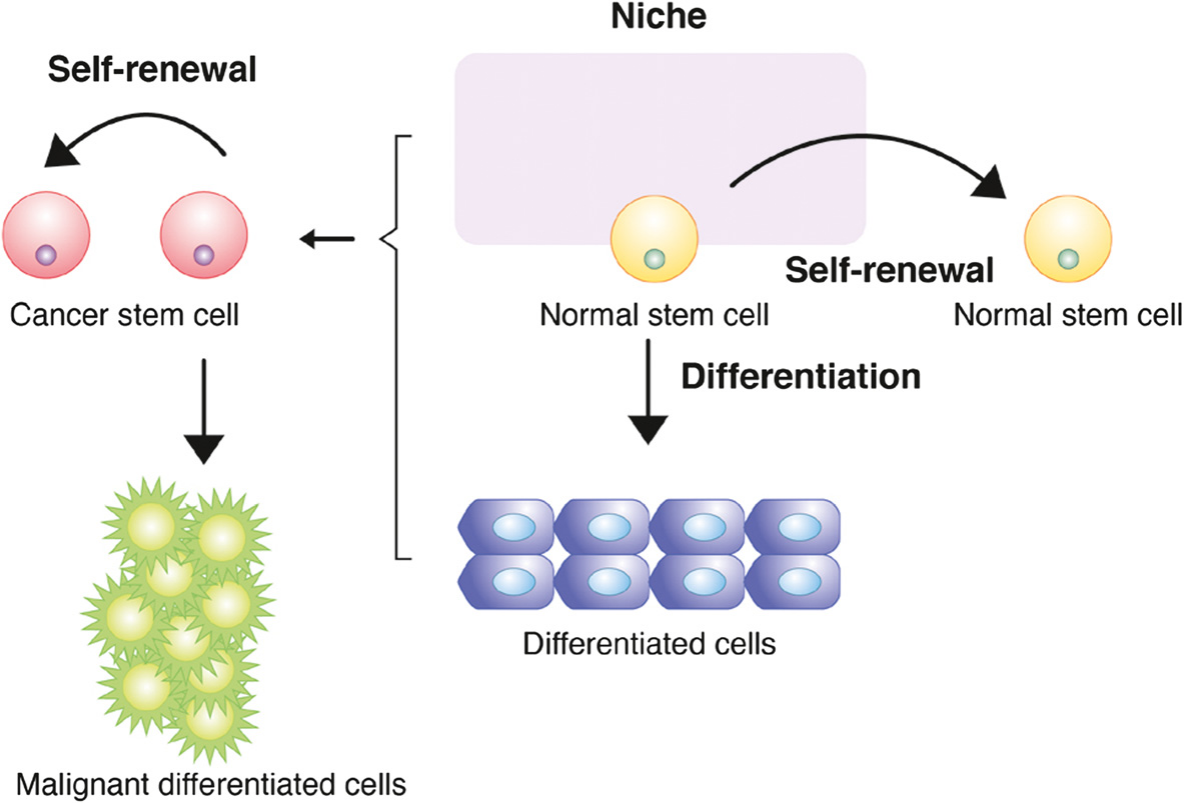
A model for self-renewal of NSCs and CSCs through asymmetric cell division. NSCs and CSCs maintain their populations in tissues or tumors through asymmetric self-renewal cell division in which one daughter cell possesses stem cell properties and the other undergoes differentiation. Through this mechanism, stem cells achieve the production of both self-renewing and differentiating cells in a single cell division. The two types of stem cells are thought to share molecular mechanisms that control asymmetric self-renewal cell division. Generally, asymmetric cell division is regulated by two types of mechanisms, intrinsic and extrinsic. Modified from (Romano 2009)

During self-renewal, the potential for both proliferation and differentiation of the parental cell is retained in one or both progenies. Many genes and signaling pathways involved in the self-renewing process of normal stem cells (NSCs) were shown to be oncogenes (Shackleton 2010). CSCs in tumors maintain their population through self-renewal cell division similar to that of NSCs in tissues (Fig. 1). Both NSCs and CSCs can self-renew through asymmetric cell division in which one daughter cell possesses stem cell properties, and the other undergoes differentiation (Betschinger & Knoblich 2004; Clevers 2005; Doe & Bowerman 2001; He et al. 2009; Knoblich 2010; Morrison & Kimble 2006). Through this mechanism, a stem cell can produce both self-renewing and differentiating cells in a single cell division. However, both CSCs and NSCs sometimes expand their population under specific processes, such as development, tissue injury, and tumor growth (Doetsch et al. 2002; Kimble & White 1981; Lechler & Fuchs 2005; Morrison et al. 1997; Wright et al. 2001). They achieve this task through symmetric cell division, which generates two daughter stem cells. The balance between asymmetric and symmetric division is orchestrated by various regulators in normal tissues and tumor masses (Morrison & Kimble 2006; Al-Hajj & Clarke 2004; Clarke & Fuller 2006). Such regulators range from key signaling mediators such as Wnt, Notch, and Hedgehog to cell cycle components such as p53 and CDK inhibitors (Al-Hajj & Clarke 2004; Luo et al. 2010; Orkin & Zon 2008). These proteins play a crucial role in not only CSC survival but also other processes, such as the survival and proliferation of NSCs in hematopoietic, neural, epidermal, and intestinal tissues.

#### Self-renewal of NSCs through asymmetric cell division

Various tissues maintain their homeostasis by specifying stem cell populations that function as a reservoir of tissue-specific cell types. In order to maintain the populations at constant levels and replenish mature cells as the need arises, stem cells use asymmetric self-renewal division which produces one daughter that remains in the stem cell lineage and the other undergoing limited rounds of transit amplification and differentiation (Fuchs & Chen 2013; Rossi et al. 2007; Shepherd et al. 2007). Many lines of evidence have demonstrated asymmetric cell division of NSCs in the blood, skin, muscle, gut, and mammary gland (Lechler & Fuchs 2005; Beckmann et al. 2007; Cicalese et al. 2009; Quyn et al. 2010; Shinin et al. 2006; Wu et al. 2007). Significant similarity was observed between CSCs and NSCs in the properties and mechanisms underlying cell proliferation, survivals, and self-renewal cell division (Al-Hajj & Clarke 2004; Reya et al. 2001). For example, most of the signaling molecules critical in survival and self-renewal of NSC were also found to be important for CSC survival. These include Wnt, Sonic hedgehog (SHH), Notch, PTEN, BMI1, p53, and p21. In addition, NSCs and CSCs share the machinery that regulates asymmetric self-renewal cell division (Luo et al. 2010; Orkin & Zon 2008; Pardal et al. 2003). Studies on various cell types revealed that asymmetric cell division is regulated by two types of mechanisms, intrinsic and extrinsic (Knoblich 2008). In the intrinsic mechanism, the cell divides asymmetrically through unequal distribution of cell-fate determinants in two daughter cells. In the extrinsic mechanism, exposure of daughter cells to differential external cues is the key factor for asymmetric division.

#### Intrinsic mechanisms underlying asymmetrical cell division of NSCs

During cell division, a subpopulation of proteins, RNAs, and other macromolecules in mother cells are inherited unequally into two daughter cells (Betschinger & Knoblich 2004; Goldstein & Macara 2007; Suzuki & Ohno 2006). Unequally distributed cellular components include fate determinants that govern the fates of two daughter cells. Prior to asymmetric division, these fate determinants are differentially enriched at either of the apical or basal pole, in which the mitotic spindle apparatus and centrosomes are unequally aligned. Proteins that promote self-renewal and stemness are recruited to the spindle apparatus at the apical side which typically faces the outside of the body or the lumen of internal cavities. In contrast, differentiation-promoting factors are recruited to the mitotic spindle located at the basal side toward the basement membrane.

Par-3, Par-6, and aPKC are key polarity-determining regulators which mark the apical pole (Kaltschmidt et al. 2000; Lee et al. 2006a). Par-3 recruits Inscuteable to the apical region which, in turn, recruits Pins (Kaltschmidt et al. 2000; Lee et al. 2006a; Kraut et al. 1996). As Pins is recruited, the unequal alignment of mitotic spindle is established along the axis of apical-basal polarity, providing an infrastructure for unequal cell division (Betschinger & Knoblich 2004; Wang et al. 2007; Mauser & Prehoda 2012). The positioning of mitotic spindle correlates to the unequal sizes and molecular compositions of the centrosomes (Chia et al. 2008; Yamashita & Fuller 2008). The larger centrosome in the mother cell is inherited to the daughter stem cell, whereas the smaller centrosome is inherited to the daughter cell that undergoes differentiation in male *Drosophila* germline stem cells and in mouse embryo neural progenitors (Yamashita & Fuller 2008; Wang et al. 2009). Once mitotic spindle and centrosomes are properly positioned in an unequal manner, a set of adaptor proteins, such as Miranda and Pon, are localized to the basal side of cells (Knoblich 2010; Bonifacino 2014). These adaptors subsequently recruit differentiation-promoting fate determinants. The adaptor Miranda regulates the asymmetric segregation of key differentiation factors such as Brat (a translational repressor) and Prospero (a homeodomain transcriptional repressor) (Ikeshima-Kataoka et al. 1997; Lee et al. 2006b; Schuldt et al. 1998; Shen et al. 1997). In Miranda mutants, all three determinants are uniformly distributed in cytoplasm and segregate equally into two daughter cells (Betschinger & Knoblich 2004; Wang et al. 2007). The adaptor Pon also is enriched in the basal pole and binds to and localizes Numb, a membrane-associated protein and a negative regulator of Notch signaling (Lu et al. 1998). Through this region-specific localization of fate-determining factors, stem cells predetermine the fates of daughter cells.

During asymmetric cytokinesis, the apical daughter cell, which is larger in size, inherits self-renew-promoting factors and remains as the stem cell lineage (Barros et al. 2003; Yu et al. 2006). In contrast, the smaller, basal cell that inherits differentiation factors, such as Numb, Prospero, and Brat, undergoes differentiation (Barros et al. 2003; Yu et al. 2006). Following unequal segregation into basal daughter cells, Numb induces differentiation by inhibiting Notch which is enriched in the apical side in mother cells and segregated into daughter stem cells (Cayouette & Raff 2002; Verdi et al. 1999; Wakamatsu et al. 1999; Liu et al. 2010). The division of a Numb-deficient mutant cell results in two stem-like cells, whereas the division of a Numb-overexpressing cell generates two differentiated daughter cells (Le Borgne et al. 2005; Petersen et al. 2002; Schweisguth 2004). The transcription factors Pros and Brat, which inhibit ribosome biogenesis and cell growth, also function as differentiation-inducing fate determinants (Betschinger & Knoblich 2004; Lee et al. 2006b; Bello et al. 2006; Betschinger et al. 2006; Frank et al. 2002). Some of cell cycle regulators, such as the cyclin-dependent kinase CDC2 of the fly *Drosophila melanogaster*, were shown to be co-segregated with fate determinants into daughter cells and play a role in their fate determination (Tio et al. 2001). Aurora and Polo kinases also play a role in fate determination by inhibiting the excess self-renewal of neuroblast (Wang et al. 2007; Lee et al. 2006c; Wang et al. 2006). Mutations of either Aurora or Polo were shown to cause symmetric cell division by disturbing the asymmetric localization of fate determinants such as aPKC, Numb, Pon, and Notch.

#### Extrinsic mechanisms underlying asymmetrical cell division of NSCs

Asymmetric cell division is also influenced by the extracellular environment. Stem cells are in close contact with a special microenvironment, called the stem cell niche, which is crucial for maintaining the stem cell identity and the potential to self-renew (Li & Xie 2005). During division, stem cells ensure that only one progeny can be in contact with the stem cell niche by keeping the perpendicular orientation of their mitotic spindle to the niche surface. The progeny in contact with the stem cell niche retains self-renewal ability, while the other undergoes differentiation. Thus, in contrast to the intrinsic mechanism which usually adopts a predefined program, the environmental niche-dependent extrinsic mechanism is relatively flexible (Lechler & Fuchs 2005; Rotundo & Fambrough 1980). It has been known that the extrinsic mechanism plays a critical role for the choice of symmetric or asymmetric divisions in HSCs. HSCs mostly divide asymmetrically when cultured on the layer of osteoblastic cells but undergo symmetric cell division on the layer of stromal cells, suggesting that HSCs control self-renewal process through interaction with the environmental niche (Knoblich 2008).

#### Mechanisms underlying asymmetric cell division of CSCs

NSCs and CSCs share similarity in the mechanisms underlying proliferation, cell survival, and self-renewal process (Austin et al. 1997; Ben-Porath et al. 2008; Bhardwaj et al. 2001; Gotoh 2009; Iliopoulos et al. 2009; Iliopoulos et al. 2010; Lemischka & Moore 2003; Lessard & Sauvageau 2003; Shimono et al. 2009; Spink et al. 2000; Taipale & Beachy 2001; Zhang et al. 2003), including the role of key regulatory proteins, such as BMI1, Notch, Wnt, and SHH (Iliopoulos et al. 2010; Shimono et al. 2009). It is therefore reasonable to speculate that the similarity is extended to asymmetric division as well. Despite the technical difficulty in isolating CSCs, recent studies began to shed new lights on the mechanistic details on CSC asymmetric division.

Pine et al. (2010) employed single-cell real-time analysis to trace the inheritance of genes during cell division of maternal CD133^+^ CSC populations isolated from human lung cancer cell lines and primary tumor cells. Interestingly, the genomic DNA in maternal CSCs was found to be unequally inherited to daughter cells. Daughter cells that inherited more maternal DNA were found to remain as CSCs, whereas those that inherited less maternal DNA became differentiated cells (Pine et al. 2010). One parameter that influences asymmetric segregation appears to be the microenvironment, such as cell-to-cell and cell-environment interaction. It was suggested that asymmetric inheritance is enhanced by cell-to-cell contact, which is influenced by cell density and environmental changes, such as serum deprivation and hypoxia (Pine et al. 2010).

To date, it still remains unknown to what degree the functions and mechanisms of fate determinants and adaptors in asymmetric division of NSCs are conserved in CSCs. Rare examples of molecules known to be unequally segregated into daughter cells include midbody derivatives (MB^d^s), a group of proteins contained in a large proteinaceous organelle, called the midbody (Kuo et al. 2011). The midbody is localized in an intercellular bridge during cytokinesis and thought to play a role in maintaining the stemness of daughter stem cells. Following asymmetric division, the midbody is normally degraded by daughter cells destined to differentiation. In daughter stem cells, however, it is protected from such autophagic degradation, leading to selective accumulation of MB^d^s. As a consequence, MB^d^s are selectively inherited to daughter stem cells.

Candidate proteins implicated in asymmetric division of CSCs include Nuclear Mitotic Apparatus (NuMa), a polarity-determining factor which is normally associated with spindle in NSCs and segregated into daughter stem cells. In NSCs, NuMa plays a role in the concentration of microtubules in the mitotic spindle poles and the bundling of the mitotic spindle to centrosomes. The asymmetric segregation of NuMa was similarly observed in *Drosophila* neuroblasts (Lechler & Fuchs 2005; Izumi et al. 2006; Siller et al. 2006). This segregation pattern was found to be perturbed by the amplification of the ongogene *MYCN* which is closely associated with neuroblastoma oncogenesis, leading to symmetric segregation of NuMa (Izumi & Kaneko 2012). The asymmetric segregation of NuMa is likely to play a role in CSCs as well.

It is known that irradiation (IR) treatment reduces tumor mass but increases the relative portion of CSCs within tumors. To address the mechanism of CSC enrichment following irradiation, Gao et al. (2013) developed the cellular Potts model (CPM) of U87-MG human glioblastoma cell line, which enhances CSC-driven tumor growth. The authors found that IR treatment increased not only the ratio of CSCs in surviving cells but also the absolute number of CSCs (Gao et al. 2013). One reasonable explanation for this observation is that in response to irradiation, CSCs shifted their cell division strategy from asymmetric to symmetric one. It should be further investigated how CSCs survive chemo- and radiotherapy.

#### De-differentiation of CSCs

The model that CSCs can repopulate heterogeneous tumor cells through asymmetric cell division is based on the assumption that CSCs and non-CSCs are epigenetically stable and can thus propagate in a mutually independent manner. Another possible explanation for how the net number of CSCs remains constant over multiple generations in established cancer cell lines or in tumors is that the epigenetic status of two cell types is flexible and able to switch from one type to the other. CSCs may be able to keep their proportion in equilibrium through these dynamic interactions with nearby bulk cancer cells in the microenvironment. Indeed, a recent study showed that the levels of CSCs and non-CSCs in human breast and prostate cancer cell lines are in dynamic equilibrium, in which the proportion of two cell types remains constant over time and through many generations (Iliopoulos et al. 2011). The equilibration in a given time could be regulated by not only formation and differentiation of CSCs into non-CSCs during symmetric and asymmetric divisions but also de-differentiation of the resulting non-CSCs back to CSCs. The signals that induce de-differentiation of non-CSCs into CSCs include secretory factors, such as the cytokine IL6. The actual balance between CSCs and non-CSCs may be influenced by the concentrations of secreted molecules and their receptors (Iliopoulos et al. 2011).

## Conclusions

Despite abundant knowledge accumulated in cancer biology and remarkable advancements in clinical translation, cancer is still one of the most fatal diseases. The major challenges in cancer treatment are the limited efficacy of chemo- and radiotherapy and the recurrence of surviving cancer cells. Accumulating evidence suggests that CSCs contribute to tumor progression and recurrence. The identification of pathways and molecules that support the properties of CSCs may lead to the development of CSC-targeting therapeutic strategies that improve the efficacy of cancer treatment. Such CSC treatments may be combined with conventional therapies targeting the tumor bulk. One key property of CSCs is their ability to undergo self-renew, which is achieved through asymmetric division. Extensive studies on NSCs revealed the mechanisms of asymmetric division which involves a number of polarity and fate determinants whose spatiotemporal distribution regulates the fate of two daughter cells. Although it is technically challenging to isolate CSCs, NSCs and CSCs will prove themselves to share similarity in the mechanisms underlying asymmetric division. Continued research on the regulation of asymmetric and symmetric self-renewal division of CSCs will provide a means to target CSCs in various tumors.
